# Comparison of sequential, delayed and simultaneous resection strategies for synchronous colorectal liver metastases

**DOI:** 10.1186/s12893-020-0681-7

**Published:** 2020-01-17

**Authors:** Li-Jun Wang, Hong-Wei Wang, Ke-Min Jin, Juan Li, Bao-Cai Xing

**Affiliations:** Key laboratory of Carcinogenesis and Translational Research (Ministry of Education/Beijing), Department of Hepatopancreatobiliary Surgery Unit I, Peking University Cancer Hospital & Institute, 52 Fucheng Road, Haidian District, Beijing, 100142 China

**Keywords:** Colorectal cancer, Liver metastases, Liver resection, Prognosis

## Abstract

**Background:**

The present study aimed to compare the perioperative safety and long-term survival of patients with synchronous colorectal liver metastases undergoing sequential resection (SeR), delayed resection (DeR) and simultaneous resection (SiR).

**Methods:**

From January 2007 to December 2016, data from patients undergoing surgery at Peking University Cancer Hospital for synchronous colorectal liver metastases were retrospectively collected. The above three different surgical strategies were compared.

**Results:**

A total of 233 cases were included, with 49 in the SeR group, 98 in the DeR group and 86 in the SiR group. The incidence of severe complications was 26.7% in the SiR group, higher than that in the DeR group (11.2%, *P* = 0.007) and the SeR group (16.3%, *P* = 0.166). The overall survival at 1 and 3 years in the SeR group (93.9 and 50.1%) was lower than that in the DeR group (94.9 and 64.8%, *P* = 0.019), but not significantly different from that in the SiR group (93.0 and 55.2%, *P* = 0.378). Recurrence-free survival at 1 and 3 years in the SeR group (22.4 and 18.4%) was lower than that in the DeR group (43.9 and 24.2%, *P* = 0.033) but not significantly different from that in the SiR group (31.4 and 19.6%, *P* = 0.275). Cox multivariate analysis indicated that T4, lymph node-positive primary tumour, liver metastases > 30 mm and SiR (compared with DeR) were correlated with poor prognosis.

**Conclusion:**

Simultaneous resection has a relatively higher incidence of severe complications, and with a staged resection strategy, the prognosis of delayed resection was better than that of sequential resection.

## Background

The liver is the most common target organ of metastases from colorectal cancer. Almost 50% of the colorectal patients will develop liver metastases during the course, and 15–25% of patients already have liver metastases at the time of diagnosis of primary colorectal cancer (synchronous liver metastases) [1, 2]. Surgery is still the best treatment to achieve long-term survival or even cure in patients with colorectal liver metastases, and the 5-year survival rate after surgery is approximately 30–50% [3, 4].

Compared with metachronous liver metastases, synchronous liver metastases usually have poorer biological behaviours, and the treatment is also more complicated. Moreover, surgery for synchronous liver metastases must address both primary and metastatic lesions and can be divided into two major categories, simultaneous and staged resection. Generally, a higher proportion of patients undergo the selective staged resection of colorectal cancer and liver metastases [5, 6]. Along with the progression of surgical techniques, the proportion of patients undergoing simultaneous resection has increased over time [7, 8]. However, simultaneous resection causes surgical trauma at two sites at the same time, which may increase the risk (e.g., intraperitoneal infection, anastomotic fistula and hepatic insufficiency). As a result, the incidence of complications and mortality are higher in simultaneous resection than in staged resection [9, 10]. Therefore, not all patients are fit for simultaneous resection, and staged resection remains an important choice. Conventionally, the primary colorectal cancer lesion is first resected, followed by the liver metastases. Later, the “liver first” surgical strategy appeared [11], that is, the liver metastases were first resected, followed by the primary colorectal cancer lesion. However, regardless of the strategy, chemotherapy between the two surgeries is selectively recommended and not mandatory [12–14], for fear of chemotherapy-associated liver injury and missing the best timing for surgery due to post-chemotherapy progression. Currently, an increasing number of patients start chemotherapy before resection of the primary colorectal cancer and liver metastases [15]. However, in selective staged resection, especially in those who have received initial chemotherapy, no consensus has been reached as to whether first-stage surgery should be sequentially followed by second-stage surgery or by interval chemotherapy and then second-stage surgery. The safety and long-term survival benefits of these two strategies compared with those of simultaneous resection have yet to be determined.

The purpose of the present study was to compare the perioperative safety and long-term survival of three different strategies, namely, selective sequential resection, delayed resection or simultaneous resection in colorectal cancer with synchronous liver metastases.

## Methods

### Study design, selection of patients and grouping

From January 2007 to December 2016, data from patients undergoing surgery at the Department of Hepatobiliary Surgery at Peking University Cancer Hospital for colorectal cancer with synchronous liver metastases were retrospectively collected and reviewed. Synchronous liver metastases were defined as liver metastases detected at or before diagnosis of the primary tumour [15]. The inclusion criteria were as follows: resection of primary colorectal cancer and liver metastases was performed with curative intent, and the patient completed the treatment; the combined extrahepatic metastases were also resected; combination with lung metastases without resection were allowable if controllable with chemotherapy [16, 17]. The exclusion criteria were as follows: (1) recurrence after resection of liver metastases; (2) non-radical surgery; (3) failure to finish resection of both the colorectal cancer and liver metastases; and (4) combination with other malignancies. Among patients with initially unresectable colorectal liver metastases, those who did not undergo second-stage surgery due to failed conversional chemotherapy after the first-stage surgery were thus excluded.

The treatment strategy was determined based on multidisciplinary discussion among colorectal surgeons, hepatic surgeons, medical oncologists, radiation oncologists and radiologists. Informed consent was obtained from the patients before the treatment began. The patients were divided into three groups based on the surgical treatment strategy: sequential resection (SeR, sequential staged resection of colorectal cancer and liver metastases without interval chemotherapy); delayed resection (DeR, staged resection of colorectal cancer and liver metastases with interval chemotherapy); and simultaneous resection (SiR, single-stage resection of primary colorectal cancer and liver metastases simultaneously). Simultaneous resection was performed in patients in whom 1) the primary tumour was located in the right colon regardless of the tumour disease burden of liver metastases through one incision; 2) the tumour disease burden was not heavy, and the tumour number was less than two, if the primary tumour was located in the left colon or rectum. Staged resection was mainly used in patients with severe symptoms due to the primary lesion, with a heavy tumour burden and a tumour number greater than three, or tumours located in the rectum that required preoperative radiochemotherapy. After the first surgery, patients with resectable liver metastases were treated with sequential resection or neoadjuvant chemotherapy, and patients with unresectable liver metastases were treated with conversional chemotherapy and then evaluated for the next surgery.

The protocol was approved by the ethics committee of Peking University Cancer Hospital and confirmed to the Declaration of Helsinki. Informed consent was obtained from all patients.

### Initial assessment

Before initial treatment, all patients underwent contrast-enhanced MRI of the liver, contrast-enhanced CT of the abdominopelvic cavity or contrast-enhanced MRI of the pelvic cavity (only for rectal cancer patients) and plain CT of the chest. Patients with disease considered resectable were assigned to undergo hepatic resection with curative intent and the aim of achieving complete resection (R0) while preserving as much normal, functional liver parenchyma (with adequate vascular inflow, outflow, and biliary drainage) as possible. The normal liver parenchyma remnant volume was > 30% [18]. If the primary colorectal cancer had already been resected at another centre, the operation notes, pathology report and postoperative complications were obtained in detail. Radiological assessment was performed to exclude any signs of residual lesions.

### Surgery

Liver resection was performed via an open procedure. The tumour number and position were determined by preoperative imaging, intraoperative ultrasound and palpation. The resected scope of the liver was determined based on the tumour number and position. For lesions that were located deep in the liver parenchyma and smaller than 2 cm in diameter, combined radiofrequency ablation was selectively performed to avoid excessive loss of liver volume. The liver resection margin was usually larger than 5 mm, and a margin of 1 mm (R1 margin status) for some lesions was acceptable as long as the chemotherapy was effective. The “liver first” strategy was selectively used in patients with a heavy tumour burden or with tumours located in the rectum requiring preoperative radiochemotherapy. Portal vein ligation (PVL) was applied in patients requiring right hepatectomy or more extensive resection with an insufficient remnant liver volume. Major liver resection was defined as the resection of 3 or more hepatic segments.

### Perioperative chemotherapy

Based on the consensus, initial chemotherapy was usually recommended for patients with an asymptomatic primary lesion in our centre; some patients refused this treatment. Systemic chemotherapy regimens were oxaliplatin- and/or irinotecan-based regimens in combination with fluorouracil and leucovorin (folfox/folfiri/folfoxiri). Combined molecularly targeted agents were selectively used according to the resectability of the liver metastases and the clinical risk scores. An assessment was performed after 2 or 4 cycles of initial chemotherapy, and those fit for surgery underwent surgery as soon as possible. For advanced middle and low rectal cancer (T3/T4 and/or N+), combined radiotherapy was administered based on local staging. For staged resection, the chemotherapy regimen given between two surgeries was usually the same as the initial chemotherapy, or folfox/capox/folfiri with or without molecularly targeted agents in the absence of initial chemotherapy. Adjuvant chemotherapy was recommended regularly if the patient’s condition allowed after surgery.

### Perioperative safety

Postoperative complications were assessed by the Clavien-Dindo grading system [19]. For staged resection, the overall incidence of complications was the sum of the incidence of complications after both surgeries, and the highest grade of complications after either surgery was taken as the final grade of complications. Severe complications were defined as those of Clavien-Dindo grade 3 or above.

### Postoperative follow-up and survival analysis

A radiological assessment was performed within 1 month after resection of both the colorectal cancer and liver metastases. Later, the patients were re-examined once every 3 months. Overall survival (OS) was defined as the interval from the start of the initial treatment (surgery or chemotherapy) to the last follow-up or death. Recurrence-free survival (RFS) was defined as the interval from resection of both the colorectal cancer and liver metastases to the time of the first recurrence. The time of the last follow-up was December 2018 for all cases.

### Statistical analysis

Statistical analysis was performed using SPSS 22.0 software. Continuous variables are described by ranges, and intergroup comparisons were conducted by t-test or U test. Categorical variables are expressed as frequencies or percentages. Intragroup comparisons were performed by chi-squared test. Kaplan-Meier survival analysis was performed, and the survival curves were compared by log-rank test. Univariate and multivariate analyses were performed using Cox models to identify prognostic factors. *P* < 0.05 indicated a significant difference.

## Results

### Comparison of baseline data among the three groups

A total of 233 consecutive cases conformed to the inclusion criteria, with 49 cases in the SeR group, 98 cases in the DeR group, and 86 cases in the SiR group. A comparison of the baseline data among the three groups is shown in Table 1. In the SeR group, the proportion of patients with a primary lesion in the rectum was significantly higher than that in the other two groups (*P* < 0.05). Moreover, the median number of liver metastases in the SiR group was lower than that in the SeR group (*P* = 0.011). In addition, the three groups of patients showed no significant differences in sex, age, T or N stage of the primary lesion, liver metastatic lesion diameter, initial CEA level, or distribution in one or two lobes. The proportion of combined extrahepatic metastases was low in all groups, with no significant differences among the three groups.

**Table 1:** The patients’ demographics and tumour characteristics

Characteristics	Sequential resection (*n* = 49)	Delayed resection (*n* = 98)	Simultaneous resection (*n* = 86)	*p*^†^	*p*^‡^	*p*^§^
Age, years (range)	54 (21–78)	59 (32–77)	57 (33–82)	0.070	0.382	0.390
Sex (male/female)	33/16	65/33	45/41	0.902	0.089	0.053
Primary tumour location Colon/rectum	22/27	63/35	62/24	0.025	0.002	0.258
T stage (T1–3/T4)	38/11	65/33	67/19	0.161	0.962	0.082
N stage (N_+_ /N0)	37/12	70/28	55/31	0.600	0.166	0.278
Number of metastases	4 (1–13)	3 (1–14)	2 (1–12)	0.097	0.011	0.277
Diameter of tumour, mm (range)	25 (1–160)	28 (3–100)	25 (3–90)	0.776	0.758	0.454
Distribution Bilobar /unilobar	29/20	56/42	42/44	0.813	0.247	0.260
Initial CEA level, ng/ml (range)	40.3 (1.3–851)	20.5 (2.7–983)	36.0 (1.2–698)	0.173	0.119	0.824
Extrahepatic metastases No/yes	47/2	91/7	76/10	0.715	0.138	0.295

Values are presented as medians and ranges

^†^ Sequential resection group versus delayed resection group

^‡^ Sequential resection group versus simultaneous resection group

^§^ Delayed resection group versus simultaneous resection group

### Comparison of chemotherapy regimen, surgical strategies and postoperative complications among the three groups

A comparison of the chemotherapy regimens and surgical strategies among the three groups is shown in Table 2. Compared with the DeR group, a higher proportion of patients received initial chemotherapy in the SeR and SiR groups (*P* < 0.05). It was as high as 83.7% in the SeR group. In addition, the proportion of patients receiving initial chemotherapy with molecularly targeted agents was also higher in the SeR group (*P* < 0.05).

**Table 2:** Treatment details and postoperative outcomes in the three groups

Characteristics	Sequential resection (*n*=49)	Delayed resection (*n*=98)	Simultaneous resection (*n*=86)	*p*^†^	*p*^‡^	*p*^§^
Initial chemotherapy Yes/no	41/8	26/72	66/20	<0.001	0.340	<0.001
Biologic agents (Yes/no)	17/32	10/88	30/56	<0.001	0.982	<0.001
Adjuvant chemotherapy Yes/no	39/10	68/30	64/22	0.190	0.497	0.450
Hepatectomy Major/minor	21/28	31/67	29/57	0.180	0.290	0.763
Combined with RFA Yes/no	7/42	11/87	5/81	0.594	0.177	0.194
Hilar vascular clamp Yes/no	46/3	82/16	66/20	0.082	0.011	0.237
Blood loss, ml (range)	200 (80-1400)	200 (70-1000)	200 (100-3200)	0.709	0.132	0.024
Differentiation of metastases				0.762	0.594	0.127
Well/Moderate/Poor	3/39/7	7/81/10	6/62/18			
Margin status R0/R1	39/10	81/17	74/12	0.651	0.329	0.528
Any morbidity				0.514	0.382	0.016
Grade 0	29	67	44			
Grade 1-2	12	20	19			
Grade 3-4	8	11	23			
Overall morbidity, n (%)	20 (40.8)	31 (31.6)	42 (48.8)	0.270	0.369	0.017
Cumulative major complications, n (%)	8 (16.3)	11 (11.2)	23 (26.7)	0.385	0.166	0.007

Values are presented as medians and ranges.

†Sequential resection group versus delayed resection group

‡Sequential resection group versus simultaneous resection group

§Delayed resection group versus simultaneous resection group

In the DeR group, the median number of chemotherapy cycles between the two surgeries was 4 (1, 25), and the median interval was 20.7 (10.1, 77.2) weeks. In the SeR group, the median interval was 5.7 (3.4, 14) weeks between the two surgeries. There were no significant differences in the proportion of patients receiving adjuvant chemotherapy among the three groups. Except for the higher proportion of portal occlusion in the SeR group, the three groups showed no significant differences in the use of extensive liver resection or combined radiofrequency ablation, intraoperative blood loss or postoperative resection margin status. A total of 34 patients in the SeR group and 6 patients in the DeR group underwent treatment with the “liver first” strategy. Only 4 patients underwent the PVL procedure, and no ALPPS (associating liver partition and portal vein ligation for staged hepatectomy) procedures were performed in any group.

Regarding postoperative safety, the perioperative mortality rate was 0 in all groups. The incidence of overall complications was 48.8% in the SiR group, higher than that in the DeR group (31.6%, *P* = 0.017) and the SeR group (40.8%, *P* = 0.369). The incidence of severe postoperative complications was 26.7% in the SiR group, which was higher than that in the DeR group (11.2%, *P* = 0.007) and higher but not significantly higher than that in the SeR group (16.3%, *P* = 0.166). The SeR and DeR groups showed no significant difference in the overall incidence of postoperative complications or the incidence of severe complications. The details of the complications and the Clavien-Dindo grades are shown in Table 2 and Additional file 1: Table S1.

### Survival analysis

**Fig. 1:**
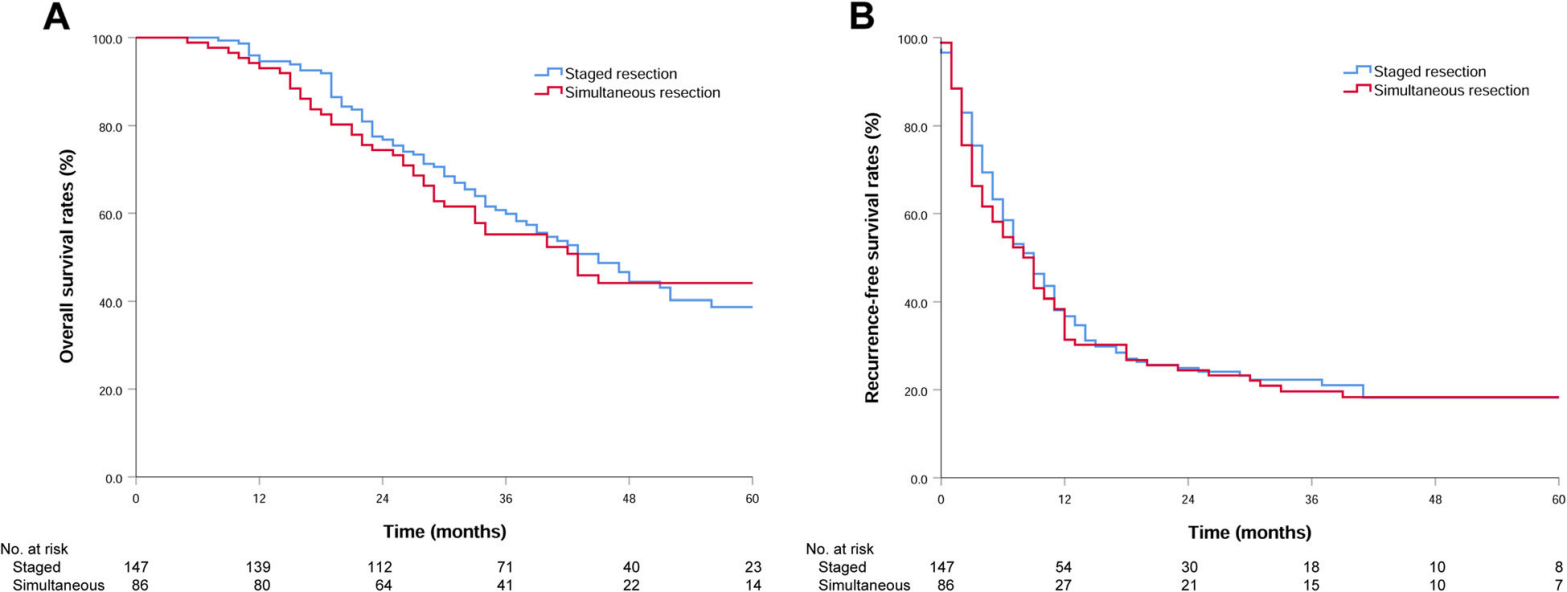
**a** Overall survival of patients who underwent staged resection or simultaneous resection; *P* = 0.887. **b** Recurrence-free survival of patients who underwent staged resection or simultaneous resection; *P* = 0.774

**Fig. 2:**
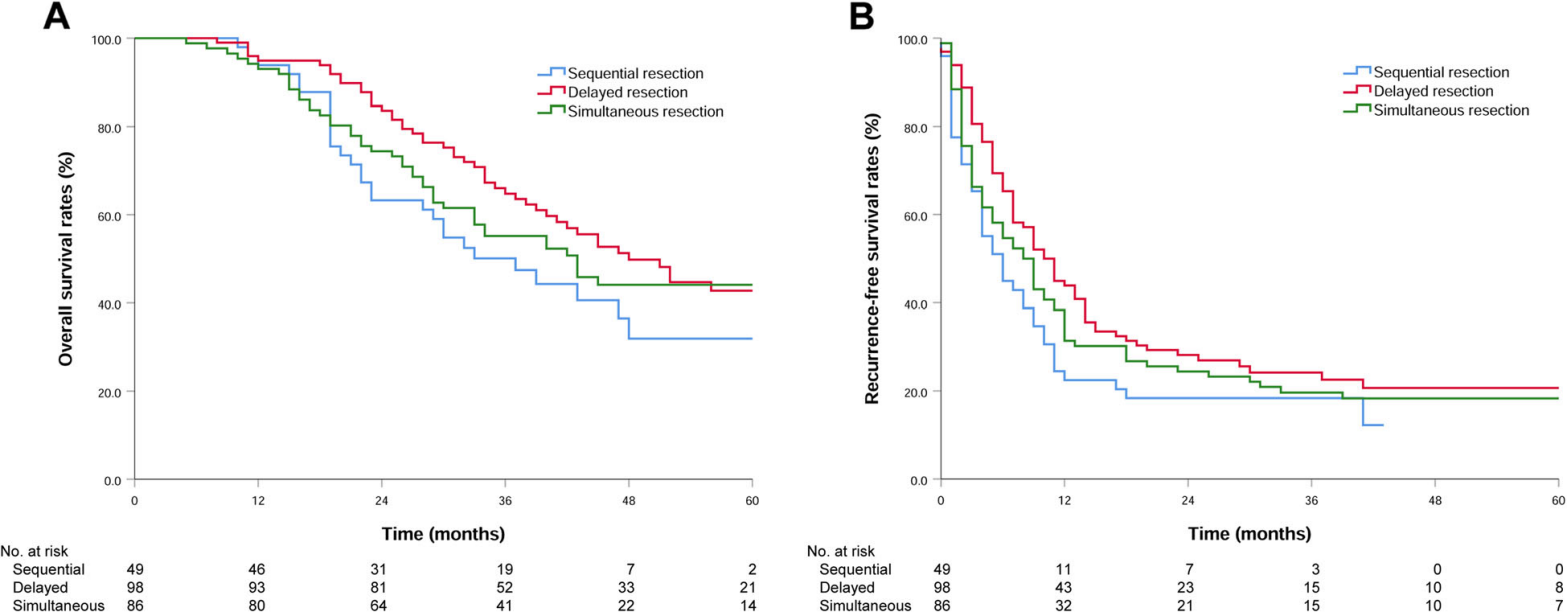
**a** Overall survival of patients who underwent sequential resection, delayed resection or simultaneous resection; *P* = 0.019 (SeR vs. DeR), *P* = 0.254 (SeR vs. SiR), *P* = 0.378 (DeR vs. SiR). **b** Recurrence-free survival of patients who underwent sequential resection, delayed resection or simultaneous resection; *P* = 0.033 (SeR vs. DeR), *P* = 0.275 (SeR vs. SiR), *P* = 0.269 (DeR vs. SiR)

There was no significant difference in the median OS (45 vs. 43 months, *P* = 0.887) or RFS (9 vs. 8 months, *P* = 0.714) between patients undergoing simultaneous resection and staged resection (Fig. 1). The 1-year and 3-year survival rates were 93.9 and 50.1% in the SeR group, 94.9 and 64.8% in the DeR group, and 93.0 and 55.2% in the SiR group, respectively. The median OS in the SeR group was lower than that in the DeR group (37 vs. 48 months, *P* = 0.019), but it was not significantly different from that in the SiR group (37 vs. 43 months, *P* = 0.378). The 1-year and 3-year recurrence-free survival rates were 22.4 and 18.4% in the SeR group, 43.9 and 24.2% in the DeR group, and 31.4 and 19.6% in the SiR group, respectively. The RFS in the SeR group was also lower than that in the DeR group (6 vs. 10 months, *P* = 0.033), but the difference was not statistically significant compared with that in the SiR group (6 vs. 8 months, *P* = 0.275) (Fig. 2).

### Univariate and multivariate analyses of overall survival

Univariate analysis was performed using the Cox regression model to identify the factors influencing OS (Table 3). The results show that the T and N stage of the primary lesion, diameter of liver metastatic lesions, resection strategies and adjuvant chemotherapy were correlated with OS. Other imbalanced factors in baseline and treatment, such as the position of the primary lesion, number of liver metastases, administration of initial chemotherapy, and use of molecular target drugs, did not affect OS. Cox multivariate analysis indicated that a stage T4 lesion, lymph node-positive primary tumour, tumour size > 30 mm and selective sequential resection (relative to delayed resection) were correlated with poor prognosis (Table 4).

**Table 3:** Factors affecting overall survival after resection on Cox univariate analysis

	Number	Hazard ratio	*P*
Sex (male vs. female)	143/90	0.812 (0.568, 1.159)	0.251
Age (> 60 vs. ≤60)	80/153	0.872 (0.597, 1.272)	0.476
Primary tumour location (rectum vs. colon)	86/147	0.886 (0.615, 1.276)	0.516
Primary tumour T stage (T4 vs. T1–3)	63/170	1.210 (1.072, 1.366)	0.002
Primary tumour node status (N_+_ vs. N0)	162/71	1.596 (1.064, 2.395)	0.024
Number of liver metastases (> 4 vs. ≤4)	70/163	1.290 (0.888, 1.876)	0.181
Extent of liver metastases (bilobar vs. unilobar)	127/106	1.338 (0.936, 1.912)	0.110
Size of liver metastases (> 30 vs. ≤30 mm)	85/148	1.700 (1.196, 2.415)	0.003
Extent of hepatectomy (major vs. minor)	81/152	1.114 (0.775, 1.602)	0.559
Combined with RFA (yes vs. no)	23/210	0.588 (0.274, 1.261)	0.172
Margin status (R1 vs. R0)	39/194	1.321 (0.838, 2.082)	0.231
Initial chemotherapy (yes vs. no)	133/100	0.940 (0.661, 1.337)	0.731
Biologic agents used in initial chemotherapy (yes vs. no)	57/176	0.931 (0.752, 1.153)	0.511
Surgical strategy			
Sequential resection	49	1	
Delayed resection	98	0.612 (0.388, 0.967)	0.035
Simultaneous resection	86	0.735 (0.463, 1.168)	0.193
Adjuvant chemotherapy (yes vs. no)	171/62	0.683 (0.464, 1.005)	0.053

**Table 4:** Factors affecting overall survival after resection on Cox multivariate analysis

	Number	Hazard ratio	*P*
Primary tumour T stage (T4 vs. T1–3)	63/170	1.930 (1.318, 2.825)	0.001
Primary tumour node status (N_+_ vs. N0)	162/71	1.585 (1.050, 2.393)	0.028
Size of liver metastases (> 30 vs. ≤30 mm)	85/148	1.537 (1.077, 2.194)	0.018
Surgical strategy			
Sequential resection	49	1	
Delayed resection	98	0.534 (0.335, 0.850)	0.008
Simultaneous resection	86	0.813 (0.510, 1.295)	0.383
Adjuvant chemotherapy (yes vs. no)	171/62	0.735 (0.494, 1.091)	0.127

## Discussion

The treatment of colorectal cancer with synchronous liver metastases requires resection of both the colorectal cancer and liver metastases. The symptoms and position of the primary lesion and resectability of the liver metastases must be taken into consideration. In addition, whether and when radiotherapy should be administered need to be considered for advanced middle and low rectal cancer. Therefore, there are no uniform standards for the clinical treatment of colorectal cancer with synchronous liver metastases.

Whether simultaneous resection is equally as safe as staged resection is a matter of debate [20]. Along with the progression of surgical technology, selective simultaneous resection is becoming increasingly applied. It has been reported that simultaneous resection does not necessarily increase the incidence of postoperative complications compared with staged resection [21]. However, it should be noted that most of the patients who underwent simultaneous resection were screened for the location of the primary lesion and/or the extent and difficulty of hepatic metastasis resection, especially those requiring extensive liver resection and/or Miles surgery for rectal cancer [22, 23]. There have been no prospective, randomized, controlled studies to answer this question definitely. At the first visit, the treatment centre and expertise of physicians in the treatment of liver metastases vary, and coordination between colorectal and hepatic surgeons is usually needed for simultaneous resection. For this reason, not all patients will be chosen for simultaneous resection. In our retrospective study, patient screening was also performed to ensure postoperative safety and recovery, and selection bias did exist. However, the results show that after screening, the overall incidence of postoperative complications and the incidence of severe complications were still higher in patients undergoing simultaneous resection than those undergoing staged resection. Apparently, if all patients underwent simultaneous dissection without screening and discrimination, more severe complications or even perioperative death might have occurred. Therefore, the staged resection strategy does have the benefit of avoiding the superposition of complications and severe complications.

Typically, patients with synchronous colorectal liver metastases are treated with initial primary colorectal cancer resection followed by 2–3 months of chemotherapy. After which, resection of the liver metastases will be performed only for those patients without interval disease progression. A previous review pointed out that this paradigm should be reconsidered since it may be not suitable for all patients [24]. Several studies showed that there was no significant difference regarding survival between patients receiving simultaneous resections and staged resections [10, 25]. This was also identified in a recent study [26]. In this study, the OS and RFS of patients undergoing simultaneous resection and staged resection did not differ significantly, and the OS was superior to that reported in those receiving palliative chemotherapy. This indicates that either strategy is reasonable and effective. However, not all patients are fit for simultaneous resection concerning the safety of simultaneous colorectal resection and major hepatic resection we mentioned above. Therefore, a staged resection strategy remains an important choice. For the staged resection, the traditional procedure is to remove the primary colorectal cancer first, and then liver metastasis will be resected in a second surgery. Nowadays, the emergence of the new “liver first” strategy appears without comprising survival results [11]. However, regardless of the strategy, whether chemotherapy should be administered between the two sequential surgeries is under debate. The survival results of patients receiving delayed resection strategy have been reported in a few studies [14, 27]. However, the sample sizes were relatively small, and the survival of patients receiving delayed resection compared to sequential resection were not involved. So, this still needs to be investigated further. A published international expert consensus [15] noted that for colorectal cancer with synchronous liver metastases without acute symptoms related to the primary lesion, systemic chemotherapy is recommended as the preferred choice. At present, an increasing number of patients receive chemotherapy before resection of either the primary colorectal cancer or liver metastases. For those who have already received chemotherapy and are scheduled for staged resection, whether chemotherapy should be given between the two surgeries is our major concern. In the present study, most of the patients in SeR group (41/49) had received initial chemotherapy, and some were even treated by molecularly targeted agents. Patients who did not receive initial chemotherapy were those who had no heavy tumour burden and a poor tolerance or refused initial chemotherapy. However, the survival analysis indicated that the median OS of patients undergoing sequential resection was lower than that of those undergoing delayed resection. Much to our surprise, according to the multivariate survival analysis, whether initial chemotherapy was administered did not affect OS, while chemotherapy administered between the two surgeries was an independent risk factor. In the baseline comparison, the SeR group had the highest proportion of patients with rectal cancer. This is because middle and low rectal cancer usually needs to be treated by synchronous radiotherapy, and the interval between the end of radiotherapy and rectal surgery is 6–8 weeks [28, 29]. For these patients, the staged strategy of resected liver metastases first and the primary rectal cancer second can be adopted. If chemotherapy is given between the two surgeries, the waiting period may be too long for second-stage resection. Oedema caused by radiotherapy may make resection of the rectal cancer very difficult, and sequential resection seems to be the only choice left. However, multivariate survival analysis using the Cox model indicated that the position of the primary tumour did not affect OS either. Although there were some imbalanced factors in the baseline data and treatment regimens between the two groups, the median OS in the SeR group was still lower than that in the DeR group after correcting for biasing factors. Another major concern is whether the tumour will progress after the first-stage surgery with the administration of chemotherapy instead of immediate second-stage surgery, which makes further surgery impossible. In the present study, after excluding patients with an initially unresectable tumour or failed to conversion chemotherapy, only 2 patients in the DeR group progressed during chemotherapy between the two surgeries, which made second-stage surgery impossible (2/100). Therefore, there is no need for excessive concern regarding tumour progression. We believe that regardless of whether initial chemotherapy is administered, it is preferable to add chemotherapy after the first-stage surgery in selective staged resection. Although it may increase the risk of chemotherapy-associated liver injury, cautious evaluation indicates that the risk is controllable for second-stage surgery and that the incidence of postoperative complications does not increase.

The survival benefits of delayed resection were higher than those of sequential resection, probably because of the following advantages of administering chemotherapy between the two surgeries: (1) the inflammation caused by the first-stage surgery may promote the spread of tumour cells [30], and chemotherapy between the two surgeries can control potential micro-metastases; (2) chemotherapy can cause further shrinkage and necrosis of the tumour, thus achieving tumour regression [31] and improving the prognosis; (3) patients are screened based on biological behaviour [32, 33] and observed for some time after chemotherapy to determine the best timing for second-stage surgery after the lesion stabilizes. This is conducive to avoiding early postoperative recurrence. The poor prognosis of patients undergoing sequential resection may also be attributed to the longer interval between resection of the primary colorectal cancer and liver metastases. Resection of either the primary colorectal cancer or liver metastases is highly traumatic. It usually takes approximately 3 to 4 weeks before the patient’s physical strength is sufficiently improved for the next surgery. However, the patient may need to wait for the arrangement of the next surgery without the protection of chemotherapy. Since the present study adopted a retrospective design, there was the problem of mismatching baseline information. Given differences in the physical status, local symptoms due to the primary lesion, referral system and level of the first visited centre, not all patients could receive treatment based on high-level multidisciplinary team (MDT) decisions. Therefore, there were no uniform standards for the choice of initial treatment. In addition, some patients were treated in the Department of Medical Oncology or in other centres, so complete, detailed records of specific adverse events or chemotherapy-induced liver injury pathological scores cannot be obtained. Moreover, the small sample size would influence the results. In the future, a prospective, randomized, controlled study will be performed to obtain more reliable conclusions.

## Conclusion

Simultaneous resection has a relatively higher incidence of severe complications, and with a staged resection strategy, the prognosis of delayed resection was better than that of sequential resection.

## Supplementary information

**Additional file 1: Table S1**. Details of postoperative complications in the three groups.

## Data Availability

The datasets generated and/or analyzed during the current study are not publicly available due to protecting individual patient privacy but are available from the corresponding author on reasonable request.

## Abbreviations

CI: Confidence interval
CRLM: Colorectal liver metastases;
DeR: Staged resection of colorectal cancer and liver metastases with interval chemotherapy
MDT: Multidisciplinary team
OS: Overall survival
RFS: Recurrence free survival
SeR: Sequential staged resection of colorectal cancer and liver metastases without interval chemotherapy
SiR: Single-stage resection of primary colorectal cancer and liver metastases simultaneously

## References

[1] Fong Y, Cohen AM, Fortner JG, Enker WE, Turnbull AD, Coit DG, Marrero AM, Prasad M, Blumgart LH, Brennan MF (1997). Liver resection for colorectal metastases. J Clin Oncol.

[2] Hayashi M, Inoue Y, Komeda K, Shimizu T, Asakuma M, Hirokawa F, Miyamoto Y, Okuda J, Takeshita A, Shibayama Y (2010). Clinicopathological analysis of recurrence patterns and prognostic factors for survival after hepatectomy for colorectal liver metastasis. BMC Surg.

[3] Kanas GP, Taylor A, Primrose JN, Langeberg WJ, Kelsh MA, Mowat FS, Alexander DD, Choti MA, Poston G (2012). Survival after liver resection in metastatic colorectal cancer: review and meta-analysis of prognostic factors. Clin Epidemiol.

[4] Nordlinger B, Sorbye H, Glimelius B, Poston GJ, Schlag PM, Rougier P, Bechstein WO, Primrose JN, Walpole ET, Finch-Jones M (2013). Perioperative FOLFOX4 chemotherapy and surgery versus surgery alone for resectable liver metastases from colorectal cancer (EORTC 40983): long-term results of a randomised, controlled, phase 3 trial. Lancet Oncol.

[5] Weber JC, Bachellier P, Oussoultzoglou E, Jaeck D (2003). Simultaneous resection of colorectal primary tumour and synchronous liver metastases. Br J Surg.

[6] Belghiti J (1990). Synchronous and resectable hepatic metastases of colorectal cancer: should there be a minimum delay before hepatic resection?. Ann Chir.

[7] Chua HK, Sondenaa K, Tsiotos GG, Larson DR, Wolff BG, Nagorney DM (2004). Concurrent vs. staged colectomy and hepatectomy for primary colorectal cancer with synchronous hepatic metastases. Dis Colon Rectum.

[8] Reddy SK, Pawlik TM, Zorzi D, Gleisner AL, Ribero D, Assumpcao L, Barbas AS, Abdalla EK, Choti MA, Vauthey JN (2007). Simultaneous resections of colorectal cancer and synchronous liver metastases: a multi-institutional analysis. Ann Surg Oncol.

[9] Bolton JS, Fuhrman GM (2000). Survival after resection of multiple bilobar hepatic metastases from colorectal carcinoma. Ann Surg.

[10] Tanaka K, Shimada H, Matsuo K, Nagano Y, Endo I, Sekido H, Togo S (2004). Outcome after simultaneous colorectal and hepatic resection for colorectal cancer with synchronous metastases. Surgery.

[11] Mentha G, Majno PE, Andres A, Rubbia-Brandt L, Morel P, Roth AD (2006). Neoadjuvant chemotherapy and resection of advanced synchronous liver metastases before treatment of the colorectal primary. Br J Surg.

[12] Vassiliou I, Arkadopoulos N, Theodosopoulos T, Fragulidis G, Marinis A, Kondi-Paphiti A, Samanides L, Polydorou A, Gennatas C, Voros D (2007). Surgical approaches of resectable synchronous colorectal liver metastases: timing considerations. World J Gastroenterol.

[13] Brouquet A, Mortenson MM, Vauthey JN, Rodriguez-Bigas MA, Overman MJ, Chang GJ, Kopetz S, Garrett C, Curley SA, Abdalla EK (2010). Surgical strategies for synchronous colorectal liver metastases in 156 consecutive patients: classic, combined or reverse strategy?. J Am Coll Surg.

[14] Lambert LA, Colacchio TA, Barth RJ (2000). Interval hepatic resection of colorectal metastases improves patient selection. Arch Surg.

[15] Adam R, de Gramont A, Figueras J, Kokudo N, Kunstlinger F, Loyer E, Poston G, Rougier P, Rubbia-Brandt L, Sobrero A (2015). Managing synchronous liver metastases from colorectal cancer: a multidisciplinary international consensus. Cancer Treat Rev.

[16] Mise Y, Kopetz S, Mehran RJ, Aloia TA, Conrad C, Brudvik KW, Taggart MW, Vauthey JN (2015). Is complete liver resection without resection of synchronous lung metastases justified?. Ann Surg Oncol.

[17] Andres A, Mentha G, Adam R, Gerstel E, Skipenko OG, Barroso E, Lopez-Ben S, Hubert C, Majno PE, Toso C (2015). Surgical management of patients with colorectal cancer and simultaneous liver and lung metastases. Br J Surg.

[18] Van Cutsem E, Cervantes A, Adam R, Sobrero A, Van Krieken JH, Aderka D, Aranda Aguilar E, Bardelli A, Benson A, Bodoky G (2016). ESMO consensus guidelines for the management of patients with metastatic colorectal cancer. Ann Oncol.

[19] Dindo D, Demartines N, Clavien PA (2004). Classification of surgical complications: a new proposal with evaluation in a cohort of 6336 patients and results of a survey. Ann Surg.

[20] Siriwardena AK, Mason JM, Mullamitha S, Hancock HC, Jegatheeswaran S (2014). Management of colorectal cancer presenting with synchronous liver metastases. Nat Rev Clin Oncol.

[21] Yoshioka R, Hasegawa K, Mise Y, Oba M, Aoki T, Sakamoto Y, Sugawara Y, Sunami E, Watanabe T, Kokudo N (2014). Evaluation of the safety and efficacy of simultaneous resection of primary colorectal cancer and synchronous colorectal liver metastases. Surgery.

[22] Silberhumer GR, Paty PB, Temple LK, Araujo RL, Denton B, Gonen M, Nash GM, Allen PJ, DeMatteo RP, Guillem J (2015). Simultaneous resection for rectal cancer with synchronous liver metastasis is a safe procedure. Am J Surg.

[23] Farid SG, Aldouri A, Morris-Stiff G, Khan AZ, Toogood GJ, Lodge JP, Prasad KR (2010). Correlation between postoperative infective complications and long-term outcomes after hepatic resection for colorectal liver metastasis. Ann Surg.

[24] Reddy SK, Barbas AS, Clary BM (2009). Synchronous colorectal liver metastases: is it time to reconsider traditional paradigms of management?. Ann Surg Oncol.

[25] Thelen A, Jonas S, Benckert C, Spinelli A, Lopez-Hanninen E, Rudolph B, Neumann U, Neuhaus P (2007). Simultaneous versus staged liver resection of synchronous liver metastases from colorectal cancer. Int J Color Dis.

[26] Strowitzki MJ, Schmidt T, Keppler U, Ritter AS, Mahmoud S, Klose J, Mihaljevic AL, Schneider M, Buchler MW, Ulrich AB (2017). Influence of neoadjuvant chemotherapy on resection of primary colorectal liver metastases: a propensity score analysis. J Surg Oncol.

[27] Yoshidome H, Kimura F, Shimizu H, Ohtsuka M, Kato A, Yoshitomi H, Furukawa K, Mitsuhashi N, Takeuchi D, Iida A (2008). Interval period tumor progression: does delayed hepatectomy detect occult metastases in synchronous colorectal liver metastases?. J Gastrointest Surg.

[28] Sauer R, Becker H, Hohenberger W, Rodel C, Wittekind C, Fietkau R, Martus P, Tschmelitsch J, Hager E, Hess CF (2004). Preoperative versus postoperative chemoradiotherapy for rectal cancer. N Engl J Med.

[29] Schmoll HJ, Van Cutsem E, Stein A, Valentini V, Glimelius B, Haustermans K, Nordlinger B, van de Velde CJ, Balmana J, Regula J (2012). ESMO consensus guidelines for management of patients with colon and rectal cancer. A personalized approach to clinical decision making. Ann Oncol.

[30] Govaert KM, Jongen JMJ, Kranenburg O, Borel Rinkes IHM (2017). Surgery-induced tumor growth in (metastatic) colorectal cancer. Surg Oncol.

[31] Blazer DG, Kishi Y, Maru DM, Kopetz S, Chun YS, Overman MJ, Fogelman D, Eng C, Chang DZ, Wang H (2008). Pathologic response to preoperative chemotherapy: a new outcome end point after resection of hepatic colorectal metastases. J Clin Oncol.

[32] Leonard GD, Brenner B, Kemeny NE (2005). Neoadjuvant chemotherapy before liver resection for patients with unresectable liver metastases from colorectal carcinoma. J Clin Oncol.

[33] Conrad C, You N, Vauthey JN (2013). In patients with colorectal liver metastases, can we still rely on number to define treatment and outcome?. Oncology (Williston Park).

