# Supplementary material for: Comparison of sequential, delayed and simultaneous resection strategies for synchronous colorectal liver metastases
Source: BMC Surg. 2020 Jan 17;20:16. doi: 10.1186/s12893-020-0681-7 (PMC6969459; doi:10.1186/s12893-020-0681-7)
Supplement: Supplementary file 1 — Additional file 1: Table S1. Details of postoperative complications in the three groups. [file 12893_2020_681_MOESM1_ESM.docx]

Table S1 Details of postoperative complications in the three groups

| Complications | Sequential | Delayed | Simultaneous |
| --- | --- | --- | --- |
|  | resection | resection | resection |
|  | (n=49) | (n=98) | (n=86) |
| Bile leakage | 1 | 3 | 0 |
| Liver failure | 2 | 3 | 2 |
| Abdominal infection | 4 | 1 | 3 |
| Fever | 3 | 2 | 7 |
| Blood transfusion requirement | 2 | 7 | 5 |
| Wound infection | 0 | 1 | 1 |
| Anastomotic leakage | 2 | 1 | 2 |
| Percutaneous drainage requirement | 5 | 7 | 11 |
| Bleeding | 0 | 1 | 4 |
| Others | 1 | 4 | 3 |

Values are presented as numbers.
